# Carbonyl-Containing Solid Polymer Electrolyte Host Materials: Conduction and Coordination in Polyketone, Polyester, and Polycarbonate Systems

**DOI:** 10.1021/acs.macromol.2c01683

**Published:** 2022-12-07

**Authors:** Therese Eriksson, Harish Gudla, Yumehiro Manabe, Tomoki Yoneda, Daniel Friesen, Chao Zhang, Yasuhide Inokuma, Daniel Brandell, Jonas Mindemark

**Affiliations:** † Department of Chemistry − Ångström Laboratory, Uppsala University, Box 538, SE-751 21Uppsala, Sweden; ‡ Division of Applied Chemistry, Faculty of Engineering, Hokkaido University, Kita 13 Nishi 8 Kita-ku, Sapporo, Hokkaido060-8628, Japan

## Abstract

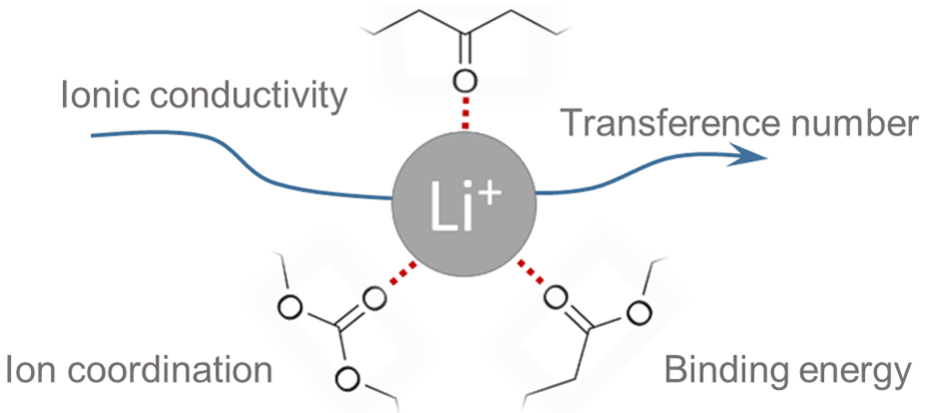

Research on solid polymer electrolytes (SPEs) is now moving beyond the realm of polyethers that have dominated the field for several decades. A promising alternative group of candidates for SPE host materials is carbonyl-containing polymers. In this work, SPE properties of three different types of carbonyl-coordinating polymers are compared: polycarbonates, polyesters, and polyketones. The investigated polymers were chosen to be as structurally similar as possible, with only the functional group being different, thereby giving direct insights into the role of the noncoordinating main-chain oxygens. As revealed by experimental measurements as well as molecular dynamics simulations, the polyketone possesses the lowest glass transition temperature, but the ion transport is limited by a high degree of crystallinity. The polycarbonate, on the other hand, displays a relatively low coordination strength but is instead limited by its low molecular flexibility. The polyester performs generally as an intermediate between the other two, which is reasonable when considering its structural relation to the alternatives. This work demonstrates that local changes in the coordinating environment of carbonyl-containing polymers can have a large effect on the overall ion conduction, thereby also showing that desired transport properties can be achieved by fine-tuning the polymer chemistry of carbonyl-containing systems.

## Introduction

Solid polymer electrolytes (SPEs) have been researched since the 1970s and nowadays mainly with the aim to have them as a safer alternative to the organic liquid electrolyte in lithium-ion batteries.^1−4^ In the bulk part of this research work, the focus has been on ether-based polymers, primarily poly(ethylene oxide) (PEO).^5,6^ In recent years, however, several other polymer host materials have become popular as alternatives to PEO. Examples are polynitriles, polyalcohols, and polyamines, but also polymers containing a carbonyl group as the coordinating unit.^7^ In this context, polycarbonates and polyesters have been quite extensively studied,^8−11^ while polyketones emerged only recently.^12^

For SPEs, the ion transport and coordination are key properties. The strength of the coordination depends on the polymer structure and the type of functional group. PEO is known to have a high coordination strength to lithium and can thereby easily dissolve lithium salts, but at the same time this also hinders the migration of lithium ions, thereby generating a low cation transference number.^7,13^ To attain a higher transference number, a host material with a weaker coordination strength is desired, for example carbonyl-containing polymers such as polyketones, polyesters, or polycarbonates.

We recently showed how the coordination and transport in a polyester–polycarbonate copolymer system were dependent on the ester-to-carbonate ratio in the polymer, where a higher coordination strength was seen with more ester groups in the system.^14^ It was experimentally observed that the polyester system had a higher coordination number and computationally that the electrostatic force between the carbonyl group and the lithium ion was stronger in the ester system compared to the carbonate system. The pronounced differences observed between these different carbonyl groups, depending on the presence of noncoordinating alkoxy oxygen atoms, suggest that these differences may be further pronounced if extending the comparison to a system devoid of alkoxy oxygen atoms, i.e., a polyketone.

Polyketones are structurally similar to polyesters and polycarbonates, but their preparation is synthetically more challenging compared to the relatively simple polycondensation and ring-opening polymerization that can be used to synthesize polyesters and polycarbonates, which has so far largely prevented their use in polymer electrolytes.^12^ To complete the set of carbonyl-containing polymers, the polyketone poly(1-oxoheptamethylene) (POHM) is here introduced together with the structurally analogous poly(ε-caprolactone) (PCL) and poly(tetramethylene carbonate) (PTeMC). With the same length of the repeating unit, these facilitate a direct comparison of coordination and transport properties between all three carbonyl-coordinating polymers while limiting the influence of steric effects. Despite the general understanding that the carbonyl oxygens are the coordinating oxygens in these types of polymers,^7^ the presence of one or two alkoxy oxygens in the main chain of polyesters and polycarbonates may affect the coordination strength of the coordinating carbonyl oxygen, and thereby ultimately also the transport properties. This effect is explored in this work both experimentally and computationally, incorporating molecular dynamics (MD) simulations to provide a more realistic description of the local coordination environment in polymeric systems.

## Materials and Methods

### Materials

Chemicals were acquired from commercial sources and, unless stated otherwise, used as received. ε-Caprolactone (gift from Perstorp AB) was distilled under reduced pressure over CaH_2_ before use. Lithium bis(trifluoromethanesulfonyl)imide (LiTFSI; BASF) was dried in a vacuum oven at 120 °C for 48 h before use. After drying, all handling and electrolyte preparation was done in an argon-filled glovebox.

### Synthesis of Poly(1-oxoheptamethylene)

**Scheme 1:**
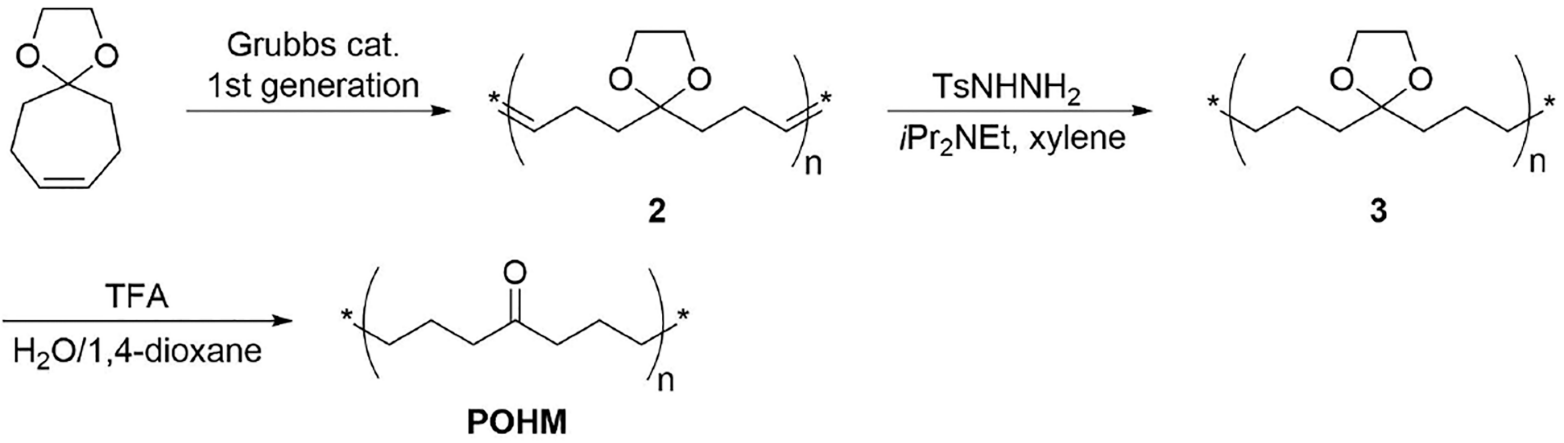
Reaction Scheme for the Syntheses of POHM

POHM was synthesized according to the reported literature^15^ from 1,4-dioxaspiro[4,6]undec-8-ene (monomer) in three steps with slight modifications (see the SI for details) (Scheme 1). The *M*_n_ was approximately 30 000 g/mol with *Đ* = 1.7.

### Synthesis of Poly(ε-caprolactone)

The PCL was synthesized through ring-opening polymerization similarly to what was described for PTMC by Rosenwinkel et al.^13^ In short, a stainless steel reactor was loaded with 4.2 g (37 mmol) of distilled ε-CL, 7.4 μL (7.4 μmol) of 1 M Sn(Oct)_2_ solution in anhydrous toluene, and 10 μL (0.14 mmol) of 1,3-propanediol in an argon-filled glovebox. The closed reactor was heated at 130 °C for 72 h for polymerization. *M*_n_ (GPC, THF): 49 500 g/mol, *Đ* = 1.58.

### Synthesis of Poly(tetramethylene carbonate)

Dimethyl carbonate and 1,4-butanediol were added to a Schlenk flask in a 2:1 molar ratio, together with a catalytic amount of K_2_CO_3_. The solution was kept under a nitrogen atmosphere at 130 °C for 4 h before vacuum was applied and the temperature was raised to 180 °C. The solution was left stirring overnight. The polymer was purified by dissolving it in dichloromethane and precipitating in methanol, before being dried over P_2_O_5_ under vacuum at 45 °C for several days. The procedure was a revised version of that described by Meabe et al.^16^ as well as Sun and Kuckling^17^ with the catalyst replaced by K_2_CO_3_. ^1^H NMR (400 MHz, CDCl_3_): δ (ppm) = 1.75 (t, −CH_2_–, poly), 3.76 (m, −CH_3_ end), 4.14 (t, −CH_2_–O–, poly). *M*_n_ (GPC, THF): 26 600 g/mol, *Đ* = 1.65.

### Polymer Electrolyte Fabrication

POHM and PTeMC were dried under vacuum at 45 °C for a minimum of 3 days before introduction to the glovebox. PCL was synthesized and kept in an inert atmosphere and required no further drying. PCL and PTeMC electrolytes were cast by dissolving the polymer and 25 or 40 wt % LiTFSI in anhydrous acetonitrile and then removing the solvent in a vacuum oven at 30 °C for 20 h while the vacuum was ramped from 200 mbar to 1 mbar, followed by 60 °C for 40 h at 1 mbar. The same procedure was done for the POHM electrolytes, with the exception that the solvent used was cyclopentanone and that a higher temperature (110 °C) was needed to dissolve the polymer. The complete removal of solvent was assured by IR and NMR spectroscopy. The PTeMC and POHM films were hot-pressed at a temperature around their melting point (60 and 160 °C, respectively) to create homogeneous films after casting.

### Structural and Thermal Characterization

The resulting formation of POHM and its purity was confirmed by NMR using a 400 MHz JEOL ECZ 400S. The molecular weights for PCL and PTeMC were measured using GPC with an Agilent Technologies 1260 Infinity. For POHM, the molecular weight was estimated from GPC on a precursor (see SI and Figure S4). To study the thermal properties, a Mettler Toledo DSC 3+ was used to obtain differential scanning calorimetry (DSC) data. The samples were run on a cool–heat–cool–heat cycle at a rate of 10 °C min^–1^ between −100 and 200 °C. FT-IR spectra were obtained on a PerkinElmer Spectrum One FT-IR spectrometer. The coordination number was obtained by fitting peaks to the carbonyl stretch peak of the FT-IR spectrum and using the ratio between the area corresponding to coordinated and noncoordinated carbonyl groups together with the [Li^+^]:[C=O] ratio of the sample.

### Electrochemical Characterization

The total ionic conductivity was measured on polymer films sandwiched between two stainless steel blocking electrodes in a CR2025 coin cell. A Schlumberger SI 1260 Impedance/Gain-Phase Analyzer was used to measure EIS in a frequency range of 1 Hz to 10 MHz using an amplitude of 10 mV in a temperature range from room temperature (23 °C) up to 90 °C. The transference number was measured using the Bruce–Vincent method^18^ in a pouch cell setup at 55 °C using an Autolab PGSTAT30. The impedance was measured before and after polarization at 10 mV. The frequency range was 0.1 Hz to 1 MHz. Using the applied voltage (Δ*V*), steady-state current (*I*_ss_), initial current (*I*_0_), and the steady-state and initial resistances (*R*_ss_ and *R*_0_), the lithium transference number (*T*_+_) was calculated using eq 1.
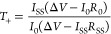
1

### Molecular Dynamics Simulations

**Figure 1:**
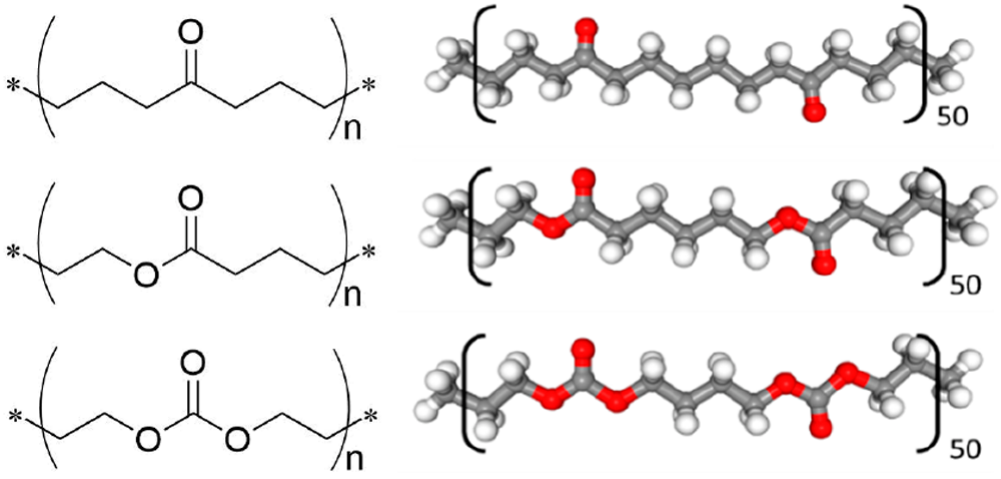
General structure with repeating unit (left) and the specific repeating molecular structure considered for molecular dynamics simulations (right) of the polyketone (POHM, top), polyester (PCL, middle), and polycarbonate (PTeMC, bottom).

The repeating units of POHM, PCL, and PTeMC considered for molecular dynamics simulations in this study are shown in Figure 1. The Generalized Amber Force Field (GAFF)^19^ parameters for a single repeating unit were generated using ambertools^20^ and acpype script.^21^ These force field parameters were used to automate the parametrization of the longer polymer chains with ∼100 repeating units and with −CH_3_ end groups. The molecular weights of the polymers used in the simulation were 11.25, 11.45, and 11.65 kg mol^–1^ for POHM, PCL, and PTeMC, respectively. The partial atomic charges for the polymer chains and TFSI were assigned using the AM1-BCC (bond charge correction) method.^22^ These charges were scaled by a factor of 0.75 for LiTFSI to reduce the ion–ion interactions and enhance the lithium-ion dynamics.^23^ The nonbonding parameters for all the atoms were taken from the standard AMBER force field. The initial configurations of the simulation boxes consisted of 10 polymer chains and 125 each of Li^+^ and TFSI ions, which corresponds to a concentration of [C=O]/[Li^+^] = 8 (∼25 wt %). These configurations were generated for all polymers using the PACKMOL package.^24^

All MD simulations were performed in GROMACS 2021,^25,26^ with a velocity Verlet integrator and a time step of 1 fs. The short-range cutoff distance for van der Waals and Coulombic interactions was set at 1.2 nm, and long-range Coulombic interactions were treated using the particle mesh Ewald summation method.^27^ The temperature and pressure of the simulations were controlled using a Bussi–Donadio–Parrinello thermostat^28^ and a Parrinello–Rahman barostat^29^ with coupling constants of 0.5 and 2 ps, respectively.

The MD protocol for equilibration of the initial configurations started with an energy minimization step using the steepest descent algorithm, an *NVT* (constant number, volume, and temperature) ensemble at 400 K for 10 ns to equilibrate temperature, and an *NPT* (constant number, pressure, and temperature) ensemble for 10 ns where *T* was varied from 400 to 1000 K and then back to 400 K to render the polymer systems completely amorphous. Thereafter, a 10 ns *NPT* run was performed at the desired temperature (400 K) to equilibrate the density. Finally, 200 ns *NPT* production runs were carried out for all the polymer electrolyte systems where the energies and trajectories were saved every 0.5 and 5 ps, respectively.

From MD simulations, the *T*_g_ can be determined by following the polymer electrolyte densities as a function of temperature. This transition from a soft/rubbery polymer state in the high-temperature range to a glassy/rigid state in the low-temperature range is then reflected as a slope change in the simulated density and temperature.^30,31^ The intersection of fitted straight lines between the low-temperature range to high-temperature range can be considered as the *T*_g_ of the simulated systems. To calculate the *T*_g_ in this way, 200 ns *NPT* annealing simulations were performed from 500 to 120 K with a step of 20 K, corresponding to a 10 ns simulation at each temperature. The first 5 ns was considered for the equilibration, and the computed densities were averaged over the rest of the trajectory.

## Results and Discussion

### Polymer Platform

To enable a fair comparison between the three polymers, the size of the repeating unit was kept the same to avoid getting extra steric or structural differences. The structure of the three polymers is seen Figure 1. By adjusting the synthesis procedures, the molecular weights were also kept in the same range of 27 000–50 000 g/mol. The synthesized polymers all turned out as semicrystalline white solids. As salt was incorporated into the polyketone, however, it turned light brown.

The length of the monomers was chosen to be the same as for PCL, as seen in Figure 1. While PCL is synthesized through a simple ring-opening polymerization reaction, the synthesis of the other two proved to be more complicated. PTeMC was synthesized through a condensation reaction of dimethyl carbonate and 1,4-butanediol similarly to that previously demonstrated.^16^ POHM was synthesized from 1,4-dioxaspiro[4,6]undec-8-ene (monomer) in three steps as seen in Scheme 1, a slightly modified route to Arrington et al.^15^ Due to the high melting point of the polyketone and its insolubility in common solvents, solvent casting was not enough to create uniform films, and therefore the films were also hot-pressed.

### Thermal Properties

**Figure 2:**
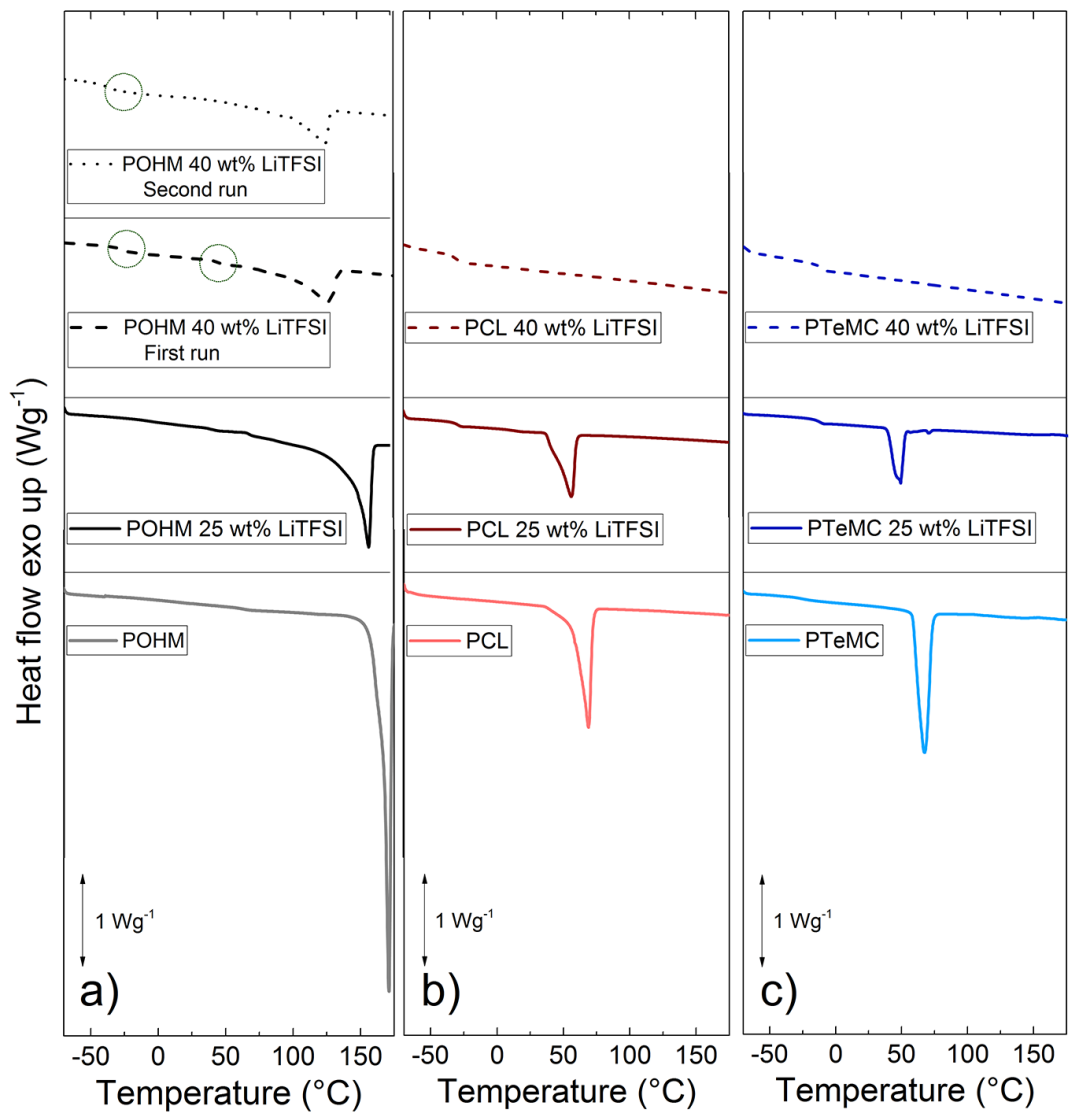
DSC data for the three polymers (a) POHM, (b) PCL, and (c) PTeMC) without and with 25 or 40 wt % LiTFSI salt. The first run is presented to show the full extent of crystallinity. Both the first and second run for POHM with 40 wt % LiTFSI are shown, and the glass transitions are circled.

DSC measurements showed that the pure polymers were all semicrystalline, with the polyketone appearing to have a higher degree of crystallinity than the other two (both with and without salt), as seen by the more pronounced melting peak in Figure 2. While PCL and PTeMC SPE films became amorphous at 40 wt % LiTFSI, the POHM remained crystalline. The enthalpy of melting for the pure POHM is around −1700 mJ/g_(polymer)_ and decreases in magnitude to around −1250 mJ/g_(polymer)_ with 25 wt % LiTFSI. With 40 wt % LiTFSI this decreases more, but 45% of the original value of the enthalpy of heating remains during the second heating step; that is, the enthalpy of melting is around −750 mJ/g_(polymer)_. For PCL these values are −840 mJ/g_(polymer)_ and −650 mJ/g_(polymer)_ and for PTeMC −860 mJ/g_(polymer)_ and −380 mJ/g_(polymer)_ for 0 and 25 wt %, respectively. As mentioned, the PCL and PTeMC samples with 40 wt % are fully amorphous. The values of exact degree of crystallinity cannot be calculated for all polymers due to the absence of a reference value for the enthalpy of melting for POHM, but the values can be used as a comparison between the different salt contents for each polymer. PCL has a *T*_g_ of −63 °C without salt, but this increases to −30 °C when salt is added, as is typically seen in SPEs due to the formation of physical cross-links.^32,33^ The same is valid for PTeMC; its *T*_g_ of −21 °C is increased to −13 °C as salt is added. The *T*_g_ is clearly higher in PTeMC than in PCL, but is still well below room temperature and thereby enabling ion transport also at ambient temperature. The melting points are very similar for PCL and PTeMC at around 50–70 °C.

The polyketone has a considerably higher melting point of around 160–170 °C. This may be seen as a positive aspect if the goal is to use the electrolyte at very high operating temperatures, but is likely to severely limit the transport properties at more moderate temperatures. Due to the high degree of crystallinity, it is also hard to detect the glass transition temperature. In the pure polymer sample, no reliable *T*_g_ can be determined. In the sample with 25 wt % LiTFSI, a *T*_g_ can be seen at around 70 °C. This is, however, probably not the true *T*_g_ of the amorphous polymer, as the very high degree of crystallinity may impact the results seen in DSC. A sample containing 40 wt % was prepared in order to decrease the degree of crystallinity and to investigate the effect of crystallinity on the properties of the polyketone. With 40 wt % LiTFSI, the degree of crystallinity is significantly decreased (see Figure 2a). This leads to the appearance of two glass transitions being visible for the first heating step: one at around −27 °C and one at 45 °C. During the second heating step, only one glass transition can be seen at −37 °C. This indicates a phase separation between more amorphous and more semicrystalline regions, where the higher *T*_g_ corresponds to the semicrystalline regions where the mobility of amorphous chains is restricted by being partially locked in crystallites. As the degree of crystallinity decreases (for example with salt addition or during the second run of a DSC measurement), the length of chain segments within the amorphous regions increases, and the *T*_g_ approaches that of a fully amorphous sample, allowing for a more accurate assessment of the segmental mobility of the polymer. This is what is seen in Figure 2a; the actual glass transition temperature is more likely close to that observed during the second run of the DSC measurement on the sample containing 40 wt % LiTFSI, namely, around −37 °C. This is the lowest glass transition out of the three polymer electrolytes, followed by around −30 °C for the PCL-based and −13 °C for the PTeMC-based electrolyte. It therefore seems to be a trend where *T*_g_ is lower with a lower number of oxygen atoms present in the main chain. The true *T*_g_ of the pure polyketone and the sample containing 25 wt % LiTFSI is likely even lower than this observed value, since the salt often increases the glass transition temperature, as mentioned previously. The results from the DSC measurements are summarized in Table 1.

**Table 1:** Summarized Results from the DSC Measurements Showing the Glass Transition Temperature (*T*_g_) and the Melting Point (*T*_m_) for the Three Polymers with and without LiTFSI and Simulated (*T*_g_) for the Three Polymers with 25 and 40 wt % LiTFSI

		experimental		simulated
		*T*_g_ (°C)	*T*_m_ (°C)	*T*_g_ (°C)
POHM	pure polymer	n.d.^a^	170	–24
	25 wt % LiTFSI	70	159	–5
	40 wt % LiTFSI	–37	125	47
PCL	pure polymer	–63	69	–3
	25 wt % LiTFSI	–30	56	20
	40 wt % LiTFSI	–31	n.d.	67
PTeMC	pure polymer	–21	66	57
	25 wt % LiTFSI	–13	49	58
	40 wt % LiTFSI	–13	n.d.	88

a n.d. = not detected.

The *T*_g_ was also estimated from the MD simulations. The averaged densities as a function of temperature are plotted in Figure S5, along with their respective fitted lines to guide the slope change. The estimated *T*_g_ values are included in Table 1. Although one can note that the simulated *T*_g_ values are about 30–70 K higher than the experimental values, they show the same trend, i.e., increasing *T*_g_ with the number of oxygens in the polymer backbone, further corroborating the effect of the different functional groups on the chain mobility of the polymers.

### Total Ionic Conductivity

**Figure 3:**
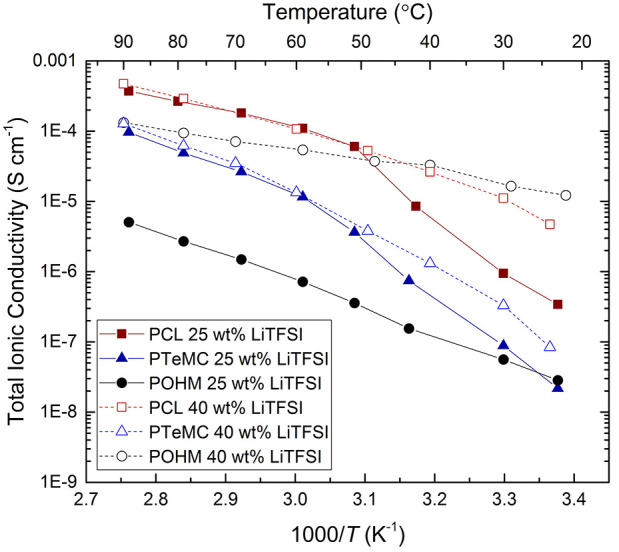
Total ionic conductivity measured through EIS for the three polymers with 25 or 40 wt % LiTFSI.

Figure 3 shows the total ionic conductivity for the three polymers from 22 °C up to 90 °C. It is clear that the semicrystallinity of the polymers influences the results, as indicated by a sharp change of slope at around 50–60 °C for both PCL and PTeMC, the temperature at which they melt. Below this point, the total ionic conductivity is limited for this same reason. The trend for PTeMC and PCL is quite similar, but the conductivity for PTeMC is about 1 order of magnitude lower over the whole range. This is in agreement with the higher *T*_g_ for the polycarbonate, which indicates that the segmental motion is more restricted.

POHM with 25 wt % LiTFSI behaves quite differently compared to the other two SPEs, because of its high melting point. The SPE is consistently below its melting point for the entire temperature range, thereby having a reduced conductivity and showing more of an Arrhenius-type behavior than a Vogel–Fulcher–Tammann-type. What is interesting to note, however, is that at room temperature the total ionic conductivities for POHM and PTeMC are comparable, despite the high degree of crystallinity of the former.

According to the classical theory on ion transport in polymer electrolytes, the conductivity should be considerably higher if the limiting crystallinity is decreased. One potential way to achieve this is by further addition of salt. This also turned out to be the case when the conductivity of a POHM sample containing 40 wt % salt was examined. The sample is still crystalline, as seen in Figure 2a, but the reduction in crystallinity indeed brings about an increase in ionic conductivity. At room temperature (22 °C), this SPE shows a remarkably high ionic conductivity of 1.2 × 10^–5^ S cm^–1^, which is higher than both the PCL and PTeMC electrolytes with 40 wt % LiTFSI. Above the melting point of PCL, however, where the POHM is still semicrystalline, the polyester has the highest conductivity also at a lower salt content. Whether the high conductivity of POHM is due to highly efficient conduction in the low-*T*_g_ amorphous parts or by contributions from a comparatively high-conductivity crystalline phase is difficult to discern. The POHM electrolytes show a more Arrhenius-like conductivity behavior over the whole temperature range. This suggests a conduction mechanism (partially) decoupled from the segmental motion of the polymer. The high ionic conductivity seen for the polyketone samples is quite remarkable as the samples have a high degree of crystallinity, a high melting point, and—as discussed above—also a high *T*_g_ for the amorphous phase within highly crystalline regions. This suggests that the crystalline phase is not fully insulating, but that it rather has a fairly high ionic conductivity even at room temperature, although still not as high as the corresponding amorphous phase. The prospect of designing a solid polymer electrolyte system with high ionic conductivity without sacrificing mechanical properties is indeed intriguing for this category of materials, with the high crystallinity acting to mechanically stabilize the material even at rather high operational temperatures.

### Ion Coordination

**Figure 4:**
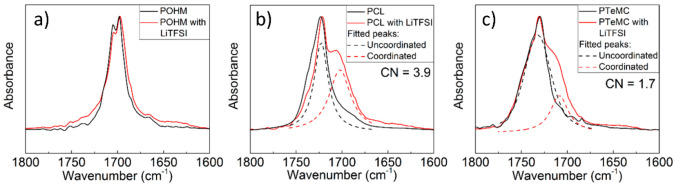
FT-IR data for the three polymers (a) POHM, (b) PCL, and (c) PTeMC, with and without 25 wt % LiTFSI salt. Dashed lines show fitted peaks for coordinated and uncoordinated carbonyl groups for PCL and PTeMC.

To further investigate how the ion transport takes place in the SPEs, the lithium coordination was investigated through FT-IR. When looking at the carbonyl stretching mode using FT-IR, it is possible to distinguish two peaks in the salt-containing samples; see Figure 4. When the carbonyl group coordinates to lithium, the peak shifts to lower wavenumbers.^34−36^ The strongest shift is seen for the PCL system, followed by the polycarbonate. The polyketone does not seem to interact much with the lithium ions, and only a small shift is seen, but it is not possible to reliably distinguish the coordinated and noncoordinated peaks due to the presence of two peaks even without the presence of LiTFSI. The peak at lower wavenumbers is overlapping with the peak for the coordinated carbonyl, and only a shift in proportion is seen between the two as LiTFSI is added. This unfortunately makes it hard to estimate the coordination number for the POHM electrolyte, while it can be straightforwardly done for PTeMC and PCL. Using the area of the fitted peaks for the two respective peaks and the [Li^+^]:[C=O] ratio of the sample, the coordination number can be calculated.^37^ The results show that PCL has a coordination number that is twice that of PTeMC (3.9 vs 1.7, as seen in Figure 4). This is consistent both in trends and in numbers with previous studies of PCL and PTMC.^14^ It therefore seems like the polyester interacts most with the lithium salt, followed by the polycarbonate and the polyketone. From previous experience, however, FT-IR is not as straightforward for polyketones as for polyesters and polycarbonates, with for example more complicated absorption bands being observed and the coordinated carbonyl group vibration shifting to higher wavenumbers instead of lower.^12^ The high crystallinity of POHM may also affect both the coordination structure and the FTIR spectrum. The combination of the complicated interpretation of the POHM spectrum and the suspiciously large difference in CN between PCL and PTeMC limits the amount of useful information that can be gleaned from these data, prompting a closer investigation of the coordination chemistry by computational means from MD simulations.

**Figure 5:**
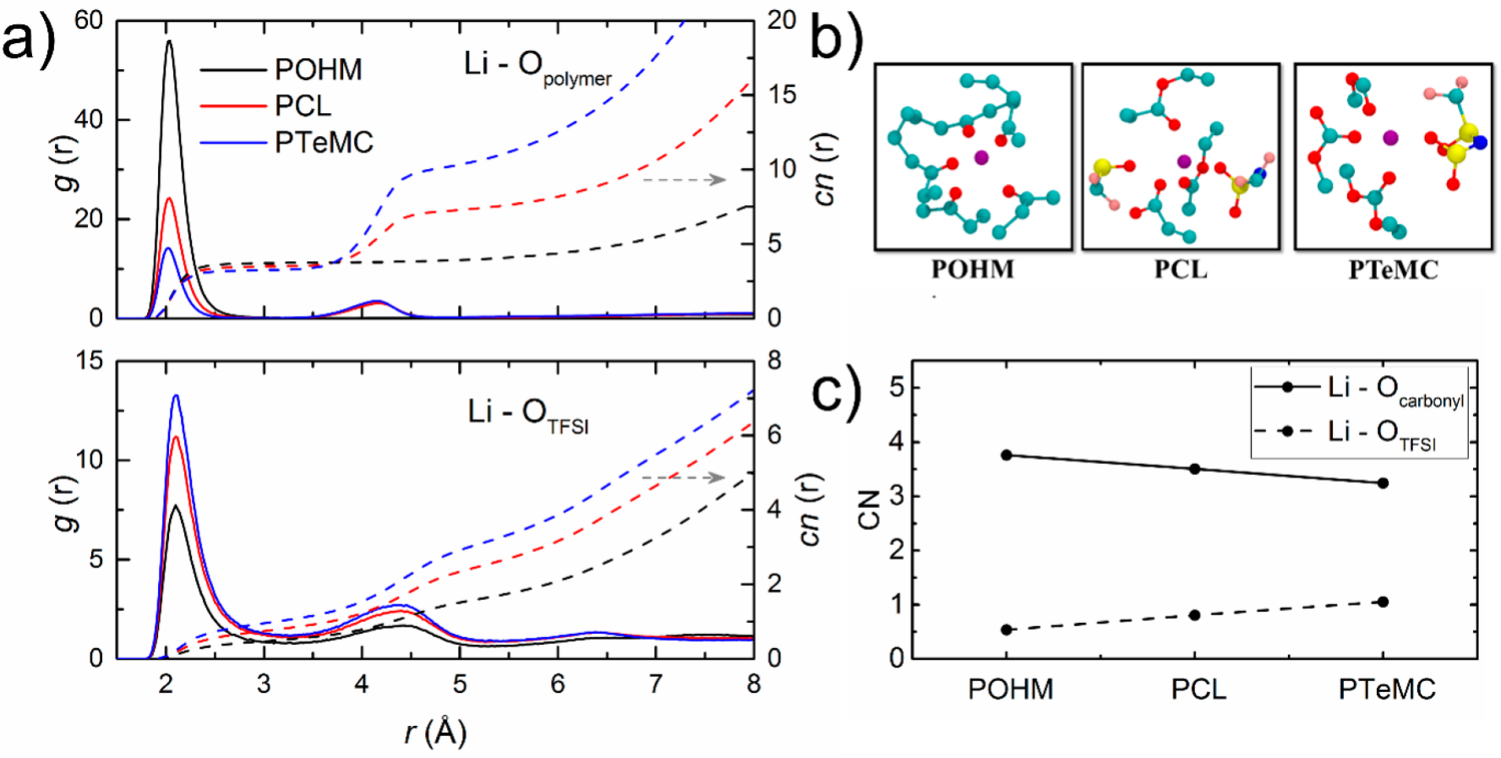
(a) Radial distribution functions *g*(*r*) and coordination number functions *cn*(*r*) for Li^+^ with O atoms from the polymer backbone and TFSI^–^ for the three polymer electrolyte systems. (b) MD snapshots of Li^+^ coordination environments within 5 Å for POHM (left), PCL (middle), and PTeMC (right). Li^+^: purple, O: red, C: cyan, N: blue, S: yellow, and F: pink. (c) Coordination number (CN) in the first coordination shell of Li–O(carbonyl) and Li–O(TFSI) for the three polymer electrolyte systems.

To study the differences in lithium-ion coordination environments, the radial distribution functions (RDF, *g*(*r*)) and coordination number functions (*cn*(*r*)) were calculated for Li^+^ with oxygen atoms from the polymer backbone and TFSI (see the SI for details). Figure 5a shows that the RDF of Li^+^ with oxygen from the polymer (Li–O_polymer_) has two peaks for PCL and PTeMC, but only one peak for POHM. This corresponds to the first peak being carbonyl oxygen atoms and the second one belonging to the main-chain oxygen atoms in the polymer backbone. This can also be confirmed from the snapshots in Figure 5b; that is, Li^+^ is only coordinated by carbonyl oxygens. Thereby, the coordination numbers calculated within the first shell of Li^+^ with oxygen from the polymer backbone will correspond to the average number of carbonyl oxygen around Li^+^. From Figure 5c, it can be seen that the coordination number (CN) of Li^+^ to carbonyl oxygen and that of Li^+^ to the oxygen in the TFSI ion for the three polymer electrolytes follow opposite trends (i.e., carbonyl oxygen coordination decreases as POHM > PCL > PTeMC, and TFSI ion coordination decreases as PTeMC > PCL > POHM), which indicates that the total coordination number of Li^+^ is similar between the electrolytes. It can be observed that the Li^+^ to carbonyl oxygen coordination number for PCL is in good agreement with the coordination number from FT-IR; however, a difference in coordination number for PTeMC can be observed. This calls into question the validity of the FT-IR measurements on the polycarbonate electrolyte and may hint toward more general quantification problems for this and possibly also related polymer systems using vibrational spectroscopy, as has previously been observed for nitrile solvents.^38^ The low Li^+^ to TFSI oxygen coordination number for POHM suggests that it actually has a better solvating ability for LiTFSI as compared to the polyester and polycarbonate, which can also be seen in snapshots from Figure 5b.

The Li^+^ transport is affected by both the local coordination environment and the coordination strength. To get an estimate of the coordination strength, the binding energy (BE) of Li^+^ in the three polymer electrolyte systems was calculated using the employed MD force field. Here, the different interactions were summarized over one MD simulation of the respective electrolyte system (i.e., polymer with salt). The differences in the interaction energies (van der Waals, *E*_vdw_, and Coulombic, *E*_Col_) of the bound state (i.e., with Li^+^) and the unbound state (with Li^+^ interactions excluded from the calculations) were calculated to estimate the BE and then averaged over all Li^+^ in the simulation.

**Figure 6:**
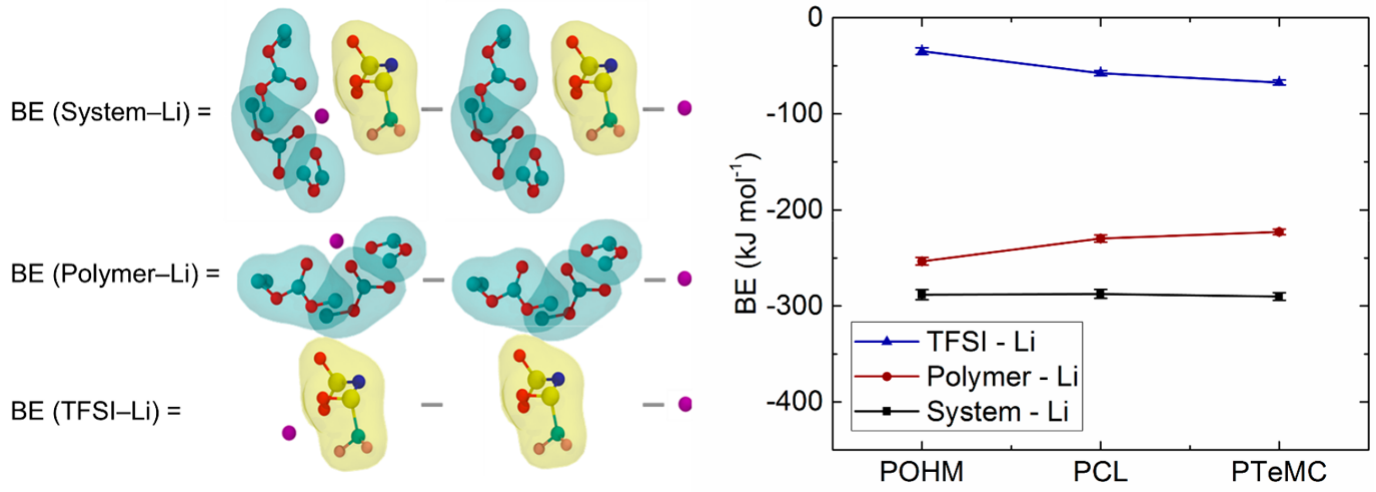
Average binding energy (BE) of all Li^+^ in the simulations with both polymer and TFSI [system–Li], as well as just with polymer [polymer–Li] and TFSI [TFSI–Li]. On the left are schematic representations of these terms. Li^+^: purple, O: red, C: cyan, N: blue, S: yellow, and F: pink. Surface colors: cyan (polymer backbone) and yellow (TFSI molecules).

As shown in Figure 6, the BE for the “system to Li^+^” gives an estimate of the binding energy of Li^+^ when interacting with all system species, i.e., both polymer and salt. In contrast, the binding energies for “polymer–Li^+^” and “TFSI–Li^+^” give the binding energies of the Li^+^ in the simulations when only the interactions with the polymer or the salt are taken into account, respectively. It has previously been shown that the binding energy of Li^+^ to various carbonate-based monomers or clusters varies from −335 to −500 kJ mol^–1^, or −451 to −643 kJ mol^–1^, using different DFT or *ab initio* calculations.^39,40^ The polymer–Li^+^ binding energy values estimated from the MD simulations were −253.5, −229.7, and −222.8 kJ mol^–1^ for POHM, PCL, and PTeMC, respectively. This difference may be expected as the electronic polarization is neglected in the classical force field used here. Nevertheless, what matters here is the relative binding energy among the three systems.

Judging from Figure 6, the BE (system–Li) for the three polymers is almost the same, with a slight increase for PTeMC. However, for the Li^+^ ion dynamics, BE (polymer–Li) is the most relevant parameter and can be seen to decrease from POHM to PCL and PTeMC. This shows that the coordination strength of the polymer to Li^+^ decreases with the extra main-chain alkoxy oxygens, while BE (TFSI–Li) follows the opposite trend. Furthermore, one can observe that the trends in BE agree with the trends in CN (Figure 5c), which is also reflected in relatively constant values for the binding energy when normalized by the coordination number (BE/CN), as shown in Figure S6. The difference in interaction strength is therefore an effect of the different number of coordinating ligands around each Li^+^ ion rather than the strength of the individual interactions.

To get further insight into the interaction between the polymer and ions, one can consider the electrostatic energy for each carbonyl group in the three systems. To calculate this, the electronic density of the carbonyl group can be estimated from their partial atomic charges given in Table 2. The charges on the oxygen and carbon of the carbonyl group (i.e., O_carbonyl_ and C_carbonyl_) increase when going from POHM to PCL, and to PTeMC; however, the increase in charge on the carbonyl *carbon* is more noticeable than the increase for the carbonyl *oxygen*. A corresponding decrease in the electron density of the carbonyl group, i.e., O_carbonyl_ + C_carbonyl_, can also be observed. To see how this decrease in electron density affects the interaction of Li^+^ with the carbonyl group, the electrostatic energy was calculated using Coulomb’s law as given in eq 2.
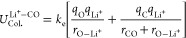
2

**Table 2:** Partial Atomic Charges of Carbonyl Oxygen (O_carbonyl_), Carbonyl Carbon (C_carbonyl_), and Alkoxy Oxygens (O_alkoxy1,2_) for Polyketone, Polyester, and Polycarbonate

atom type	POHM	PCL	PTeMC
O_carbonyl_	–0.53	–0.54	–0.58
C_carbonyl_	0.57	0.63	0.77
O_alkxoy1_		–0.45	–0.39
O_alkoxy2_			–0.39

Here, *k*_e_ is the Coulomb constant, *q*_O_, *q*_C_, and *q*_Li^+^_ are the partial charges, *r*_O–Li^+^_ is the first maximum in the RDF of Li^+^ with polymer oxygen, i.e., 2.02 Å, and *r*_CO_ is the bond length of the carbonyl group, i.e., 1.22 Å. The calculated electrostatic energies for POHM, PCL, and PTeMC were −119.83, −101.03, and −68.58 kJ mol^–1^, respectively. This shows that the electrostatic interactions of the carbonyl group with Li^+^ decrease and thus result in a decrease of coordination strength with extra alkoxy oxygens in the polymer backbone. Interestingly, this increased electrostatic interaction strength for mainly POHM but also PCL as compared to PTeMC is primarily manifested as an increase in CN rather than an increase in the strength of the individual electrostatic interactions (Figure S6).

### Transference Number

As stated earlier, the transference number is sensitive to the coordination strength between the lithium ion and the polymer. If a lithium ion is coordinated too strongly, it will decrease the transference number as the ion is trapped and not free to migrate. It could therefore be expected that the transference number should follow the trend seen in coordination numbers and binding energy, i.e., that the polyketone (with the highest polymer–ion coordination strength; see Figure 5) should have the lowest *T*_+_, followed by the polyester and the polycarbonate. This also turned out to be the case when comparing the *T*_+_ for POHM, PCL, and PTeMC, as can be seen in Table 3. For POHM, the *T*_+_ is only 0.23, while the PTeMC electrolyte has a transference number as high as 0.87. PCL has a *T*_+_ in between the other two with a value of 0.54. Thus, a trend appears in which the polymer with fewer oxygens in the functional group has a lower transference number as *T*_+_(POHM) < *T*_+_(PCL) < *T*_+_(PTeMC).

**Table 3:** Measured Transference Numbers for the Three Polymers

	*T*_+_
POHM	0.23
PCL	0.54
PTeMC	0.87

### General Discussion

The experimental results for the polyester and the polycarbonate are more reasonable to compare directly, in contrast to the polyketone, since they are more similar in terms of crystallinity and melting point. The polycarbonate has a higher glass transition temperature, which explains why the total ionic conductivity is around an order of magnitude lower than for the polyester at both salt contents. On the other hand, even though the total ionic conductivity is higher in the polyester, a large portion (about half) of the electrolytic current is anionic. The relatively low value of the PCL transference number appears to stem from the comparatively high coordination strength, making the cations less mobile. The polyketone is in this context a bit of an outlier and was difficult to characterize and compare to the other two experimentally because of its high degree of crystallinity and high melting point. POHM melts at around a 100 °C higher temperature than the other two polymers, which restricts the total ionic conductivity, at least at 25 wt % LiTFSI. At 40 wt % LiTFSI, the degree of crystallinity is lower and the ionic conductivity is therefore much higher, and is even the highest at room temperature out of the three polymers. It is quite interesting how this polymer can have such a high ionic conductivity despite still being highly crystalline. This hints at an ionic conductivity decoupled from the segmental motion in this system and that it might be possible to optimize this polyketone electrolyte to get an SPE that shows high ionic conductivity without sacrificing the mechanical properties that are generally associated with semicrystalline systems. It remains to be seen what the exact origin of this phenomenon is and whether it can be generalized to other electrolyte systems based on either polyketones or other host materials.

The results from the MD simulations complement the experimental results and show clearer trends compared to the experimental results as the polyketone in the MD data is not affected by its very high degree of crystallinity. It is therefore easier to compare the computational results for all three polymers. It is here clear that even though the structures of the polymers are similar, the extra alkoxy oxygens present in the ester and carbonate groups play a major role in not only the thermal properties but also in determining the coordinating properties. The experimental ionic conductivity for POHM is, however, in the same range as for the PTeMC electrolyte even though its *T*_g_ is the lowest, but this is likely due to the high degree of crystallinity of POHM. The lithium-ion transport will, however, always be hindered by the strong coordination (as seen mainly from the MD simulations) in the POHM system. This explains why a low *T*_+_ is seen experimentally and is also contributing to a lower total ionic conductivity. The PTeMC, on the other hand, has a high *T*_+_ due to its weaker coordination properties, which means that even though the ionic conductivity is rather low, most of the conductivity stems from the transport of lithium ions. PCL mainly performs as an approximate average between the two, which is not surprising, as the functional group is structurally an intermediate between the other two. It does, however, perform the best when it comes to ionic conductivity when looking at a reasonable salt content of 25 wt %, as it does not suffer from a too high *T*_g_ or a too high degree of crystallinity. A summary of the results and trends seen in this work is found in Table 4.

**Table 4:**
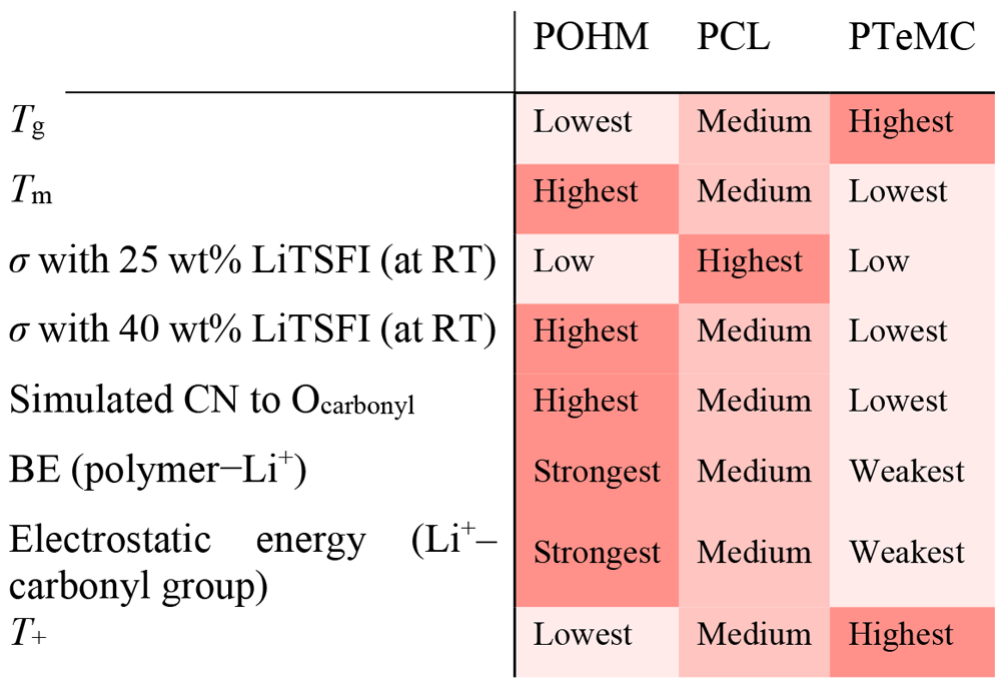
Summary and Comparison of the Results for the Polyketone, Polyester, and Polycarbonate

It is quite striking that the addition of one or two oxygens to the carbonyl group on the main polymer chain will affect the properties of the polymers to this extent, especially since the carbonyl oxygen is the main coordinating motif in all polymers. These differences change not only the physical properties of the polymer but also the lithium coordination properties. It is possible that a change in electronic density on the carbonyl group (see Table 4), as well as changes in structural rigidity that seem to come with having more oxygens in the functional group, greatly affects the properties of the polymers, considering that all other variables were kept constant.

## Conclusion

In this work, a polyketone, a polyester, and a polycarbonate of similar structure were synthesized and fabricated into SPE films with the same LiTFSI content. The electrolyte systems were also subjected to MD simulations. The functional groups of these are all carbonyl-containing, with the carbonyl group being the main lithium-coordinating motif in all three systems. Despite the similarities, it is shown that the difference in main-chain (noncoordinating) oxygens plays a significant role for several physicochemical and ion transport properties. According to the MD simulations, the glass transition temperature follows the trend POHM < PCL < PTeMC, which is reflected in the ionic conductivity. When the cation coordination number and binding energies were investigated, it was seen both computationally and experimentally that POHM possesses the highest CN and BE, followed by PCL and PTeMC. These trends in BE and CN correlate with the trends of *T*_+_; that is, when Li^+^ is more strongly bound, its movements will be restricted. This can be explained by a decrease in the magnitude of the electrostatic energy between Li^+^ and the carbonyl group when there are more alkoxy oxygens in the polymer backbone. Therefore, the local change of the functional group of carbonyl-containing polymers greatly affects the overall electrolyte properties. This suggests that one could fine-tune the polymer chemistry of this type of materials in order to optimize the corresponding ion transport properties.

## Supporting Information Available

Details of experimental procedures and simulations (PDF)
