# Supplementary material for: Carbonyl-Containing Solid Polymer Electrolyte Host Materials: Conduction and Coordination in Polyketone, Polyester, and Polycarbonate Systems
Source: Macromolecules. 2022 Dec 7;55(24):10940–9. doi: 10.1021/acs.macromol.2c01683 (PMC9798856; doi:10.1021/acs.macromol.2c01683)
Supplement: Supplementary file 1 — ma2c01683_si_001.pdf [file ma2c01683_si_001.pdf]

Supporting Information for

Carbonyl-Containing Solid Polymer Electrolyte Host Materials: Conduction and Coordination in Polyketone, Polyester and Polycarbonate Systems

Therese Eriksson[1], Harish Gudla[1], Yumehiro Manabe[2], Tomoki Yoneda[2], Daniel Friesen[1], Chao Zhang[1], Yasuhide Inokuma[2], Daniel Brandell[1], Jonas Mindemark[1]

[1] Department of Chemistry − Ångström Laboratory, Uppsala University, Box 538, SE-751 21 Uppsala, Sweden.
[2]Division of Applied Chemistry, Faculty of Engineering, Hokkaido University, Kita 13 Nishi 8 Kita-ku, Sapporo, Hokkaido 060-8628, Japan

Synthesis of polyketone

General Information

Solvents and reagents were purchased from FUJIFILM WAKO Pure Chemical Industries Ltd., TCI Co., Ltd., or Sigma-Aldrich Co., and used without further purification unless otherwise noted. All the ^1H NMR spectra were recorded using a JEOL JMN-ECS400 spectrometer. Chemical shifts are reported in parts per million (ppm) relative to residual solvent peak (δ= 7.26 ppm for ^1H in $CDCl_3$). 1,4-dioxaspiro[4,6]undec-8-ene (**1**) was prepared according to the reported procedure.[S1] Polymers **2-4** were synthesized according to the literature procedure[S1] with slight modifications.

*Ring-opening metathesis polymerization of 1,4-Dioxaspiro[4,6]undec-8-ene (**1**)*

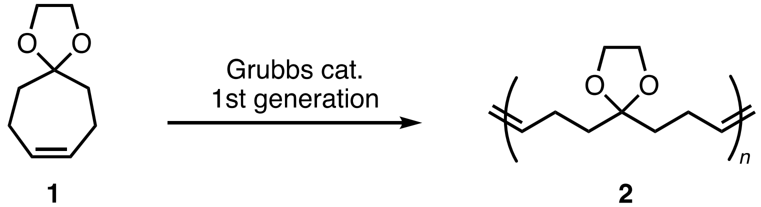

To a 50 mL two-necked flask, Grubbs catalyst first generation (107 mg, 0.130 mmol) and anhydrous, degassed dichloromethane (3.0 mL) were added under nitrogen atmosphere. The resulting purple solution was dried by blowing nitrogen gas over the solution. Afterwards, the reaction flask was cooled to 15 °C, and monomer **1**[S1] (5.98 g, 38.8 mmol) was added via syringe. After the addition, the reaction mixture was stirred, staying at 15 °C. The stirring was almost

stopped after 2 h due to the increase of viscosity of the reaction mixture. After 16 h, ethyl vinyl ether (3.0 mL) was added to the reaction mixture to deactivate the Grubbs catalyst. The reaction mixture was poured into methanol (250 mL) and the resulting sticky solid was collected by suction filtration. The solid was washed with methanol (100 mL) on a funnel. The collected solid was further dried over 2 days under vacuum to afford polymer **2** (5.19 g) as a brownish sticky solid. Although remaining monomer can be seen in the ^{1}H NMR spectrum, polymer **2** was used in the next hydrogenation step without further purification.

The ^{1}H NMR data of polymer **2** were matched with the reported literature.[S1]

^{1}H NMR (400 MHz, $CDCl_3$, 298 K): δ = 5.47 – 5.30 (m, 2H), 3.95 – 3.90 (m, 4H), 2.18 – 1.99 (m, 4H), 1.70 – 1.60 (m, 4H). See Figure S1.

*Hydrogenation of polymer **2***

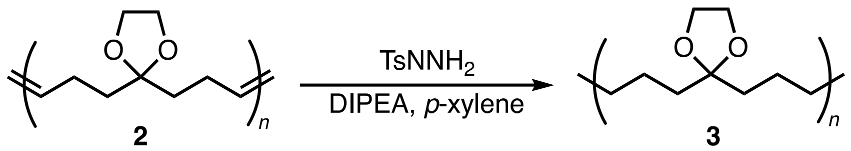

To a 1 L round-bottom flask equipped with a reflux condenser, polymer **2** (5.19 g), *p*-xylene (331 mL), *p*-toluenesulfonyl hydrazine (43.8 g, 235 mmol) and *N,N*-diisopropylethylamine (44.0 mL, 25.6 mmol) were added. The reaction mixture was refluxed for 6 h under constant stirring. After cooling to room temperature, the reaction mixture was poured into methanol (2.5 L) and was left at 0 °C overnight. The precipitated polymer was collected by suction filtration and washed with methanol (300 mL). The collected polymer was further dried under vacuum for 8 h to give polymer **3** (3.82 g, 24.4 mmol) as a colorless solid in 63% yield over two steps.

The ^{1}H NMR data of polymer **3** were matched with the reported literature.[S1]

^{1}H NMR (400 MHz, $CDCl_3$, 333 K): δ = 3.92 (s, 4H), 1.67 – 1.48 (m, 4H), 1.40 – 1.20 (m, 8H). See Figure S2.

*Deprotection of Polymer **3***

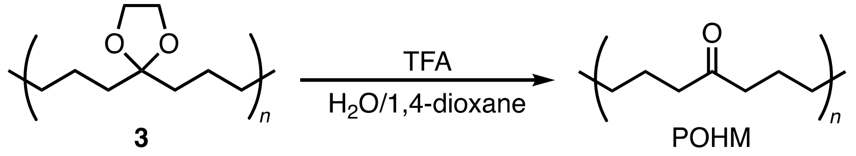

To a 1 L round-bottom flask equipped with a reflux condenser, polymer **3** (3.80 g, 24.3 mmol) and 1,4-dioxane (285 mL) were added. The reaction mixture was heated to 80 °C for 15 min to dissolve polymer **3**. To the reaction solution, water (15.2 mL) and trifluoroacetic acid (15.2 mL) were then added and the reaction mixture was stirred at 80 °C for 72 h. After cooling to room temperature, the reaction mixture was poured into methanol (2.7 L) and left at 0 °C overnight. The precipitated

polymer was collected by suction filtration and dried under vacuum for 2 days to give poly(1-oxoheptamethylene) (POHM) (2.70 g 24.1 mmol) as a colorless solid in 99% yield.
The ^{1}H NMR data of POHM were matched with the reported literature.[S1]
^{1}H NMR (400 MHz, $CDCl_3$, 333 K): δ = 2.37 (t, J = 7.3 Hz, 4H), 1.63 – 1.51 (m, 4H), 1.36 – 1.25 (m, 4H). See Figure S3.

NMR spectra

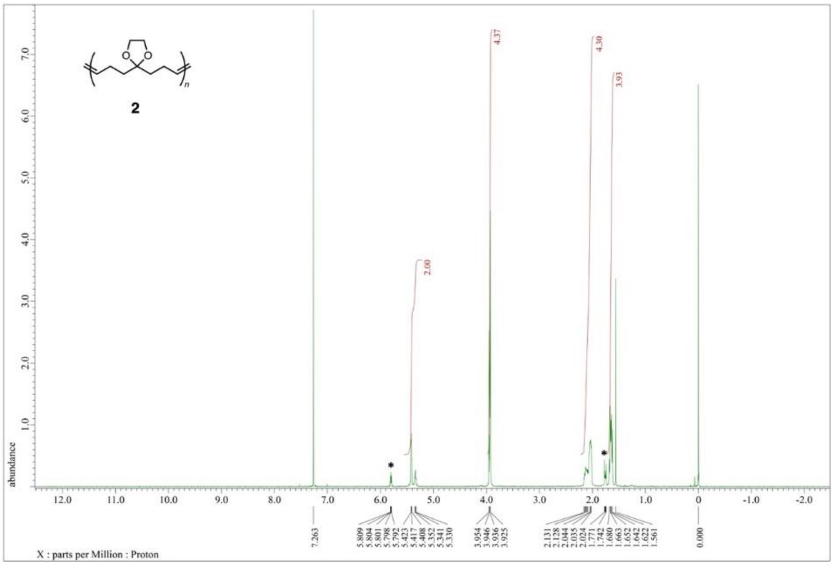

Figure S1: ^{1}H NMR spectrum of polymer **2** (400 MHz, 298 K, $CDCl_3$). Asterisk (*) indicates the residual monomer signals.

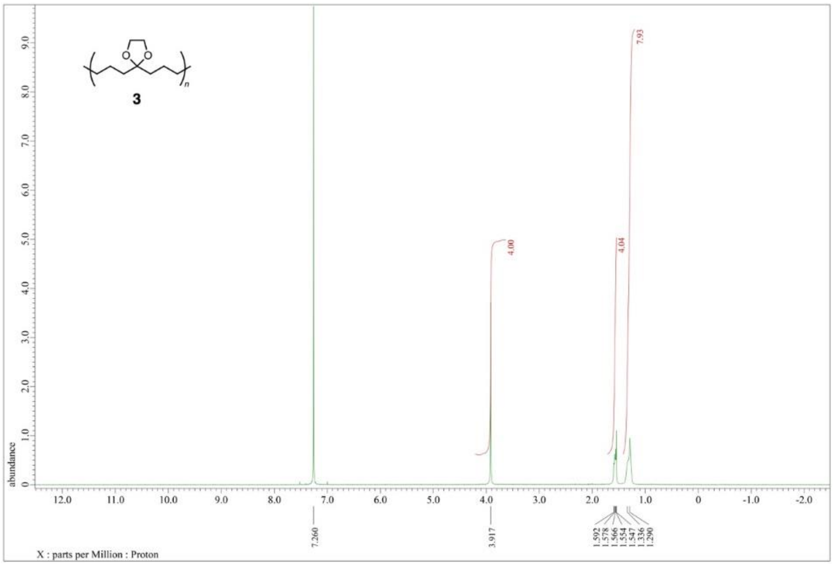

Figure S2: ^{1}H NMR spectrum of polymer **3** (400 MHz, 333 K, $CDCl_3$).

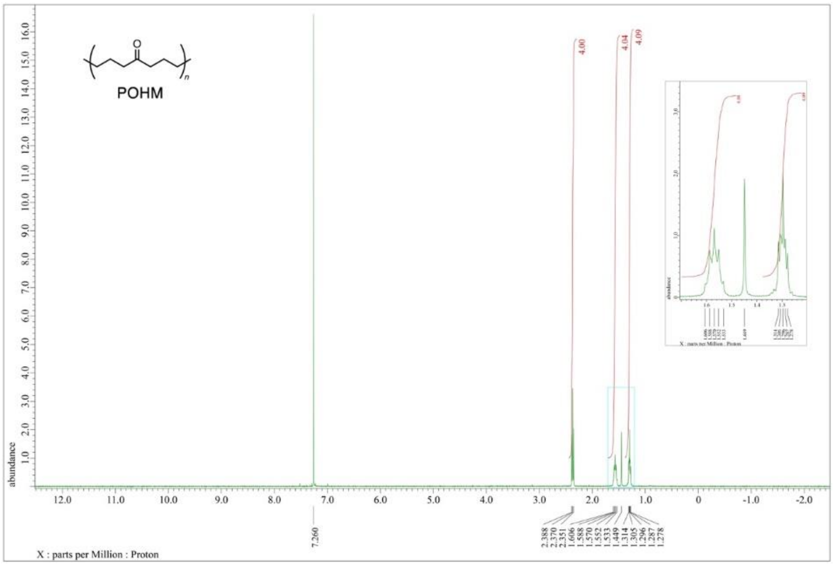

Figure S3: ^{1}H NMR spectrum of poly(1-oxoheptamethylene) (POHM) (400 MHz, 333 K, $CDCl_3$).

GPC analysis of polymer 2

Gel permeation chromatography (GPC) analysis was carried out using a JASCO PU-4180 pump, JASCO AS-4050 autosampler, and a Shodex KF-803 column equipped with JASCO CO-4060 column oven at 30 ºC. JASCO RI-4030 refractive index detector was used for the detection. Tetrahydrofuran was used as a solvent, and the flow rate was set at 0.8 mL/min. The number-average molecular weight (M_n), weight-average molecular weight (M_w) and polydispersity (M_w/M_n) were determined based on the calibration curve of polystyrene standards (Agilent InfinityLab EasiVial PS-L).

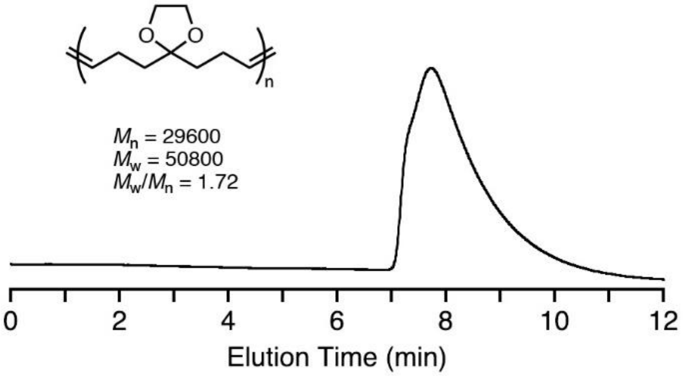

Figure S4: GPC chromatogram of polymer **2**.

MD simulations

Radial distribution functions (g(r)) and Coordination number (CN)

The radial distribution functions describe on average how the atoms are radially packed around each other. It can be defined as the ratio between the local density on a spherical shell of thickness δr at a distance r from the chosen atom and the average density. The radial distribution function $g(r)$ can be obtained by the following equations:

$$g(r) = \frac{1}{\rho N} \sum_{i} \sum_{j \neq i} \frac{\langle \delta(r_{ij} - r) \rangle}{4\pi r^2} \quad (1)$$

with ρ being the number density. The volume integral of the $g(r)$ from zero to the first minimum after then nearest neighbor peak (first coordination shell) gives the number of direct neighbors of each atom, i.e., the average coordination number of atoms throughout the simulation:

$$CN = 4\pi\rho \int_0^{r_{min}} r^2 g(r) dr. \quad (2)$$

Glass transition temperature

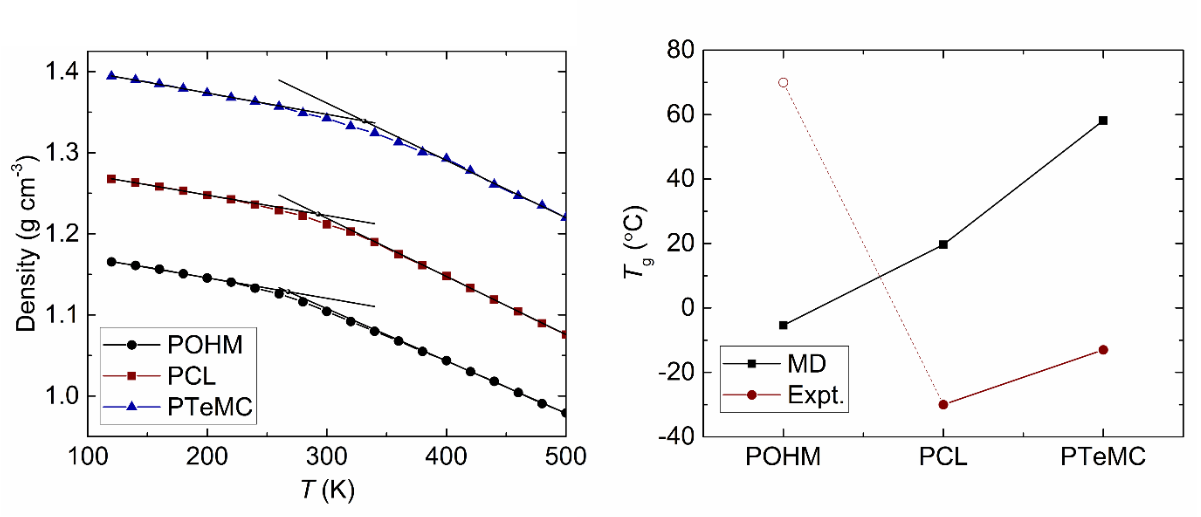

Figure S5: (left) The averaged densities of POHM, PCL, and PTMC with 25% LiTFSI salt as a function of temperature. The grey lines represent fitted straight lines in both high and low-temperature ranges and the black dot is the intersection of these lines. (right) Comparing the T_g values from experiment and MD simulations for POHM, PCL, and PTMC with 25% LiTFSI salt.

BE and CN

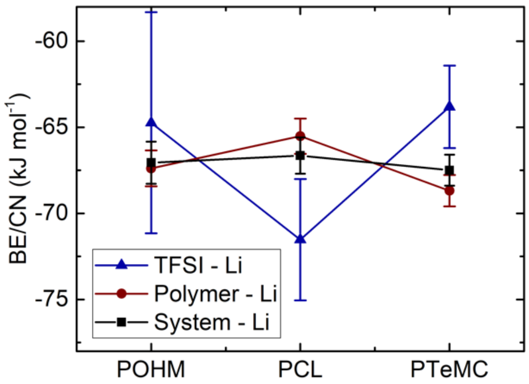

Figure S6: The ratio of average binding energy (BE) and coordination numbers (CN(Li–O_X)) of System–Li, Polymer–Li, and TFSI–Li.

References

(S1) K. J. Arrington, C. B. Murray, E. C. Smith, H. Marand, J. B. Matson, *Macromolecules* **2016**, *49*, 3655–3662.
